# Investigation of Tree Spectral Reflectance Characteristics Using a Mobile Terrestrial Line Spectrometer and Laser Scanner

**DOI:** 10.3390/s130709305

**Published:** 2013-07-19

**Authors:** Yi Lin, Eetu Puttonen, Juha Hyyppä

**Affiliations:** 1 Institute of Remote Sensing and Geographical Information Systems, Beijing Key Lab of Spatial Information Integration and Its Applications, Peking University, Beijing 100871, China; 2 Department of Remote Sensing and Photogrammetry, Finnish Geodetic Institute, Masala 02430, Finland; E-Mails: eetu.puttonen@fgi.fi (E.P.); juha.hyyppa@fgi.fi (J.H.)

**Keywords:** mobile terrestrial, line spectrometer, laser scanner, individual tree spectral reflectance, vegetation index

## Abstract

In mobile terrestrial hyperspectral imaging, individual trees often present large variations in spectral reflectance that may impact the relevant applications, but the related studies have been seldom reported. To fill this gap, this study was dedicated to investigating the spectral reflectance characteristics of individual trees with a Sensei mobile mapping system, which comprises a Specim line spectrometer and an Ibeo Lux laser scanner. The addition of the latter unit facilitates recording the structural characteristics of the target trees synchronously, and this is beneficial for revealing the characteristics of the spatial distributions of tree spectral reflectance with variations at different levels. Then, the parts of trees with relatively low-level variations can be extracted. At the same time, since it is difficult to manipulate the whole spectrum, the traditional concept of vegetation indices (VI) based on some particular spectral bands was taken into account here. Whether the assumed VIs capable of behaving consistently for the whole crown of each tree was also checked. The specific analyses were deployed based on four deciduous tree species and six kinds of VIs. The test showed that with the help of the laser scanner data, the parts of individual trees with relatively low-level variations can be located. Based on these parts, the relatively stable spectral reflectance characteristics for different tree species can be learnt.

## 1. Introduction

Accurate and comprehensive perception of individual tree properties is a scientific topic of interest, and the related techniques have recently been highlighted, particularly, by the forestry community. The reasons are that this subject is not only necessary for better understanding the various features of different tree species in precision plant management [1], but also useful for supplying more accurate sampling for estimation training in large-area forest inventories [2]. Correspondingly, a large variety of measurement instruments and data-processing approaches have been developed individually aimed at different tree properties. For example, hyperspectral imaging tends to be assumed for retrieving tree biochemical properties [3] and laser scanning is often used for reconstructing tree structural geometries [4]. Fusion of hyperspectral imaging and laser scanning has been proposed as a solution as well for the acquisition of more complete tree properties [5]. In addition, various survey platforms and mapping modes have been attempted, such as on satellites [6], airplanes [7], unmanned aerial vehicles (UAVs) [8], tripods [9], and vehicles [10]. A brief literature review shows that as an important supplementary plan to aerospace and airborne measurements, terrestrial (*i.e.*, ground-based) investigation can supply a potential way for accurately measuring tree properties.

Terrestrial hyperspectral imaging systems and terrestrial laser scanning systems have both been enthusiastically developed and attempted for fine-scale tree properties investigation. For hyperspectral imaging, the related ground-based systems have been used for such as characterization of vegetation spectral features [11]. For laser scanning, the associated static terrestrial systems have been used for such as modeling of 3D trees [12], estimating leaf area density [13], obtaining the 3D structure of tree orchards [14], and estimating forest structural attributes [15]. To fulfill the goal of comprehensive perception of object properties, static terrestrial systems that integrate hyperspectral imaging and laser scanning have also been developed. Readers interested in the corresponding endeavors can refer to [16–18], which involved establishing projection models for data fusion as well as applying the integrated systems in urban structure type characterization and rock characterization at centimeter scales, respectively. The related systems and mapping principles have the potential of supplying an optimal way for investigating tree properties [19]. However, the impact of obscuration makes it necessary to laboriously relocate the measuring device several times around a tree for its complete representation, and thus, the traditional static terrestrial approaches tend to suffer from the problem of low efficiency.

Mobile mapping [20,21], which is a research topic currently attracting quite a lot of attention, can improve measurement efficiency. Laser scanners mounted onto various mobile terrestrial platforms have been increasingly described these years, and the subject of tree properties investigation based on mobile laser scanning (MLS) has been increasingly exploited. The associated application cases involve individual tree recognition [22], assessment of the influence of foliation on laser pulse echoes [23], real-time tree-foliage surface estimation [24], multi-echoes-based crown reconstruction [25], *etc.* As regards mobile hyperspectral imaging, its implementation tends to be concisely mentioned in the specs of mobile mapping systems [20], and little research on tree spectral reflectance properties has been reported. While the feasibility of combining mobile hyperspectral imaging and laser scanning data for tree species classification has been exploited [26], the features corresponding to mobile hyperspectral imaging data was still extracted and used in a simplified mode, *i.e.*, the average of the spectra for a whole tree was used to characterize that tree. This is due to the fact the resulting spectra set for each tree has large variations. The effect of directional light scattering in different plant land surfaces has also been studied [27]. The results showed that the differences between the minimum and the maximum reflectance measured from the same target can be over hundred percent, depending on the viewing geometry. Hence, it showed that the acquired spectra would have variations of, at least, a similar order. In other words, the inherent characteristics of tree spectral reflectance in the case of mobile terrestrial hyperspectral imaging were still unclear.

Aimed at bridging this technical gap, this study was dedicated to investigating the spectral reflectance characteristics of single trees with a mobile mapping system. The system comprising a hyperspectral imaging module and a laser scanning module was preferred. The latter facilitates recording the structural characteristics of the target trees synchronously, and this can help reveal the characteristics of the spatial distributions of tree spectral reflectance with variations at different levels. In addition, since it is difficult to manipulate the whole spectra, the traditional concept of vegetation indices (VI) [28–33] based on some particular spectral bands was taken into account. Further, it was anticipated that we could find some VIs capable of showing consistent values for the whole crowns of the individual trees, and then, the associated spectral bands can stably represent the tree spectral reflectance characteristics. In consequence, the specific tasks were planned as follows: (1) testing whether the parts of the trees with relatively low-level variations can be located; (2) checking whether some VIs are capable of behaving consistently for the whole crown of each individual tree.

## 2. Methodologies

### 2.1. Mobile Terrestrial Line Spectrometer and Laser Scanner System

The Sensei mobile mapping system developed in the Finnish Geodetic Institute [34] was assumed to conduct the test campaign of tree properties investigation. Sensei comprises a number of measurement instruments, *i.e.*, an inertial measurement unit/global positioning system (IMU/GPS) attitude/location module, two laser scanners, an optical camera, a line spectrometer and a thermal camera. The Sensei system is constructed under a modular structural frame, which means that only the modules required for the specific measurement campaign need to be installed. Thus, new sensors can be easily added to Sensei. In the present study, a Specim V10H hyperspectral camera (Spectral Imaging Ltd., Oulu, Finland), an Ibeo Lux laser scanner (Ibeo Automotive Systems GmbH, Hamburg, Germany) and a NovAtel SPAN-CPT inertial navigation system (NovAtel Inc., Calgary, Canada) were integrated. The configuration of the resulting measurement system can refer to [34].

Specifically, the Specim V10H is a line spectrometer. It has an instantaneous field of view of 0.067° and a spectral resolution of 8.5 nm. Its spectral range covers from 397 nm to 1,086 nm and its opening angle of the optics is 44.4° for all of the pixels arrayed vertically in the configuration of the Sensei system. The spectrometer measures the incident light by passing it through a line-pattern diffraction grating (*i.e.*, once sampling results into a “line-set” of spectral data) to a monochromatic charge-coupled device (CCD) sensor with 659 × 493 pixels. 659 denotes the spatial grids and 493 indicates the spectral channels. Note that the spectral channels here have been averaged during data acquisition by binning the pixels corresponding to the same samplings into 123 channels to reduce the measurement noise and the amount of data to be stored and post-processed. The reflectance spectra were normalized using a Spectralon™ reference panel (Labsphere, Inc., North Sutton, NH, USA). The panel was fixed with a tilt angle of 45° onto the frame of Sensei the way that the outermost 10 pixels of the line spectrometer measured the reference spectra during every spectrum collection operation. The specific setup and the related description can refer to [26]. This ensured the results to be the relative reflectance factors *R*.

The Ibeo Lux scanner receives laser returns from four scan profiles simultaneously and it has a theoretical scan rate of up to 38,000 points/s. if only one return per laser pulse and per layer is recorded. The scanner is able to record up to three echoes per pulse. This allows it to receive echoes from the ground or buildings even which are shaded by vegetation. Its distance measurement range is from 0.3 to 200 m (50 m for targets with 10% remission), its ranging accuracy is 10 cm, and its angular resolution is 0.25°. The divergence of its laser beam is 0.8° horizontally and 0.08° vertically with respect to the scanner body in the assumed instrument configuration.

### 2.2. Test Site and Data Collection

The test site is located in Vanttila, Southern Finland (60°13′N, 24°39′E), which covers a garden and the two sides of the street leading to it for experiment. The garden consists of over 200 tree and shrub specimens representing over 20 different species. The chosen specimens are briefly planted with small distances between each other and with clear understory. In this scenario, the obscuration impacts often occurring between the target trees can be neglected. The tree species and the related numbers of trees per species are listed in Table 1. All of the four tree species are deciduous. The whole trees of the four tree species were mapped and recorded each from one side.

The campaign of data collection was conducted at the earlier September 2010. The date was in the late summer in Finland and the leaves of the trees were still green. The time was after 9 o'clock in the morning. This means that the zenith angle of sun was ∼71° from nadir. This large zenith angle alludes that the sunlight-impacting effects were serious in the hyperspectral data. The data were collected by driving along the street and the paths around and within the test garden. Thus, the scanned sides of the trees range from fully illuminated sides to shaded sides. The data were measured over a time span of 7 min and it consisted of five million laser points and 10,000 line-sets of spectral data.

### 2.3. Integration of Heterogeneous Data

Investigation of the spectral reflectance characteristics of an object based on hyperspectral imaging tends to be an ill-posed inverse problem [3] due to none of its structural information. The combination of mobile laser scanning here can supply this information. However, this does not mean it is enough, since different geometric models are assumed in their samplings and heterogeneous data is supplied. In particular when the mobile measurement mode is concerned here, even the available geometric models for static terrestrial hyperspectral imaging and laser scanning [18] cannot be employed directly. Hence, the integration of these two heterogeneous data-sets needs to transform them into the same coordinate frame with their projection geometric models considered in prior.

**Figure 1.**
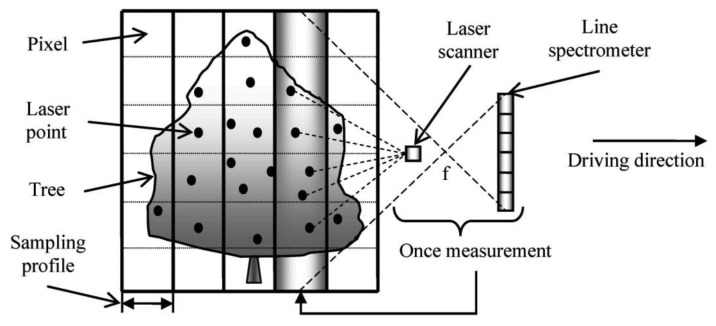
Demonstration of the basic projection geometric models of mobile terrestrial line spectrometer and laser scanner.

The basic projection geometric models of mobile terrestrial line spectrometer and laser scanner are displayed in Figure 1. Both the laser scanner and the line spectrometer used in this study conducted the measurements in a mode of sampling profile, *i.e.*, once measurement relates to an echo-comprising profile for laser scanner (termed as scan-profile) and a pixel-arrayed strip for line spectrometer (termed as spec-profile). Then, the observed space can be represented by concatenation of the parallel scan-profiles or spec-profiles individually. Within these profiles, the geometric model of laser scanner can be characterized by a “central-radiation” model, and the geometric model of line spectrometer can be featured by a central perspective projection model. The parameters of the two instruments can be learnt from their manuals and their spatial offsets can be precisely measured in prior [34].

The detailed data integration process was as follow: The IMU-derived attitudes/locations were first interpolated by using the time stamps of the laser points. Then, the point cloud data were transformed into the Sensei inertial frame coordinates. The integration of the hyperspectral imaging data and laser scanning data was fulfilled by direct georeferencing based on the post-processed attitude/position data of the IMU/GPS system. The post-processing was done through Kalman filtering using the Waypoint Inertial Explorer software and GPS base station data from Geotrim's Virtual Reference Station network. The exact information about the accuracy of the data was not available, since no control points were measured. But based on the error metrics such as the standard deviation and gap between the forward and reverse solutions, the accuracy turned out to be better than 10 cm. After the integration, possible overlapping between the spectrometer line images and the laser points was first tested in Sensei's moving direction (horizontal). Next, the laser footprint here was reckoned as a pure point with three coordinates, and the overlap test was repeated in the vertical direction for the laser points found within the pixels of each spectrometer profile. The reflectance values of every pixel located in a single laser point were normalized by division against the reference spectra measured from the Spectralon™ reference plate. Then, the processed data was saved into a new data structure of line array, in which each element serially contained the three coordinate values of the laser point and the spectrum values of the related pixel. Finally, the targeted individual trees were extracted manually from the resulting laser point clouds and hyperspectral imaging data.

### 2.4. Vegetation Indices Calculation

As shown in [26], the spectra of all the pixels for a tree were complex. Specifically, the difference between the minimum and the maximum reflectance measured from the same tree can be over hundred percents. It is hard to derive tree spectral reflectance characteristics based on the raw hyperspectral imaging data. Hence, a proper parameter is needed for assessment. The VIs [28–33] are combinations of surface reflectance at two or more spectral bands designed to highlight a particular spectral feature of vegetation. This kind of indices characterize not only tree spectral reflectance at special bands but also tree physiological or biochemical properties. Thus, six VIs [28–33] as listed in Table 2 were assumed here to investigate tree spectral reflectance characteristics.

The six VIs were chosen for illustratively assessing the spectral reflectance of the target trees. Their formulas are also listed in the Equations (1)–(6) in which *R*_i_ means the reflectance of the ith wavelength band. Note that the chosen VIs were initially proposed in the previous endeavors aimed at different measurement scales, e.g., at leaf or canopy scales. The determination of scales is generally up to the spatial resolutions of the used surveying devices. The VIs at canopy scales were proposed to deal with the cases of measurement devices with coarse resolutions. In this study, the mobile terrestrial mapping system was adjacent to the target trees, the VIs at leaf scales can also be explored. Thus, to learn tree spectral reflectance characteristics more comprehensively, the VIs at both leaf and canopy scales were calculated and compared.

(1) $$\left(R_{800}-R_{445}\right)/\left(R_{800}-R_{680}\right)$$

(2) $$\left(R_{800}-R_{445}\right)/\left(R_{680}-R_{445}\right)$$

(3) $$R_{750}/R_{550}$$

(4) $$\left(R_{800}-R_{670}\right)/\left(R_{800}+R_{670}\right)$$

(5) $$\left(R_{800}-R_{670}\right)/\left(\text{SQRT}\left(R_{800}+R_{670}\right)\right)$$

(6) $$2.5*\left(\left(R_{800}-R_{670}\right)/\left(R_{800}-\left(6*R_{670}\right)-\left(7.5*R_{475}\right)+1\right)\right)$$

### 2.5. VI Spatial Distributions for Individual Trees

The short distances between the mobile terrestrial measurement system and the target trees make it possible to calculate an amount of VI values for each individual tree. However, the values of the same VI type may differ largely for a same tree. The possible reasons lie in that the different parts of a tree have different structures, which may put different influence on sunlight reflectance. In the traditional works on hyperspectral imaging, it is hard to analyze such impacts. But in this study, the synchronous data collected by laser scanning can represent the structural features of the trees, which are helpful for quantification of the VI spatial distribution for each individual tree. This is also one of the advantages of the assumed mobile line spectrometer and laser scanner system.

The strategy for the assessment of VI spatial distributions for single trees is to partition each tree in space for consideration. This idea stemmed from the previous findings that the VIs at the edges of trees are more easily influenced [27]. Moreover, the influences at different parts of the edges of trees are dominantly decided by different factors. For example, the influences at the top parts of trees are mostly triggered by sunlight direct incidence, whereas the influences at the bottoms stem majorly from the background. Given that hyperspectral imaging records spectral reflectance mainly from tree surfaces, partitioning the trees into parts with spectra variations at different levels was manipulated based on the outer surfaces of the trees. The resulting VI spatial distributions are of significance for tree properties investigation in high accuracy.

### 2.6. Spectral Reflectance Characteristics for Different Tree Species

In addition to the assessment of VI spatial distributions for individual trees, the spectral reflectance characteristics for different tree species were also taken into account. The reasons are that different tree species generally present different structural patterns, which may result into different modes of spectral reflectance. Specifically, the assessments focused on two aspects: (1) How can the parts with spectra variations at different levels be determined for each of the tree species listed in Table 1? The resulting parts presenting different degrees of impacts are referential for choosing the appropriate parts for studying the real spectral reflectance characteristics of the trees. (2) What are the spectral reflectance characteristics for the targeted tree species? For example, different tree species correspond to different clusters in the multiple-VI-composed feature space. The derived features can be used for tree species classification.

## 3. Results and Discussions

### 3.1. Data Analysis

**Figure 2.**
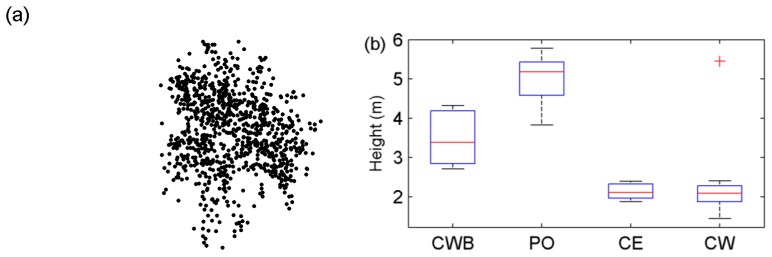
Illustration of the represented trees based on the laser scanning data (a) and the statistics of the heights of the target trees individually for the four tree species (b).

After the integration of the two heterogeneous data-sets, the data for investigation of tree spectral reflectance characteristics were acquired. Based on the mobile laser scanning data, the represented trees of the CWB specie are illustrated in an oblique view in Figure 2a. This exemplification has shown the theoretical inference that the laser scanning with low sampling densities cannot reconstruct the trees in details. Moreover, the statuses of the tree heights are listed in Figure 2b in terms of tree species.

**Figure 3.**
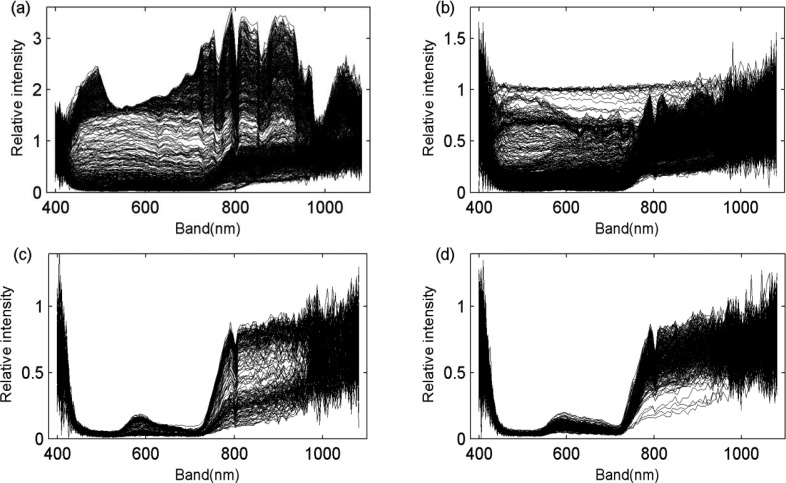
Illustration of the spectra of a tree individually for the species of (a) CWB; (b) PO; (c) CE; and (d) CW.

The spectra of the four target tree species are illustrated in Figure 3. It can be learnt that the spectra of even a single tree is divergent, far more complex than the standard spectra such as in the Vegetation Spectral Library [35]. That is, it is hard to accurately retrieve the physiological properties of trees from such hyperspectral data. However, it can be interpreted that the principal appearances of the spectra are approximate to the corresponding standard spectra, particularly for the latter two tree species. The “red edge” peculiar for vegetation can be perceived, even in the spectra of the first two tree species. As regards the large spectral variations for the bands over 800 nm in Figure 3c,d, it may be caused by the light-scattering effect in crowns becoming stronger for the short-wave infrared bands under the background of blue sky or the signal-to-noise ratio of the sensor at these wavelengths.

Note that in practical measurements, the mobile mapping data collections may cover hundreds of, even thousands of, road-side trees within ten minutes, and it is difficult to model the radiative-transfer models of the trees each by each. As illustrated by the target trees in this study, the scanned sides of the trees range from fully illuminated sides to shaded sides. Thus, this work was aimed at this outdoor scenario and attempted to find some ways in data preprocessing to handle the problem of tree spectral reflectance with large variations. As regards precise modeling of tree spectral reflectance, the works involving such as indoor measurement and radiative transfer modeling will be deployed by the authors in the next-step work.

### 3.2. Vegetation Indices Calculation

**Figure 4.**
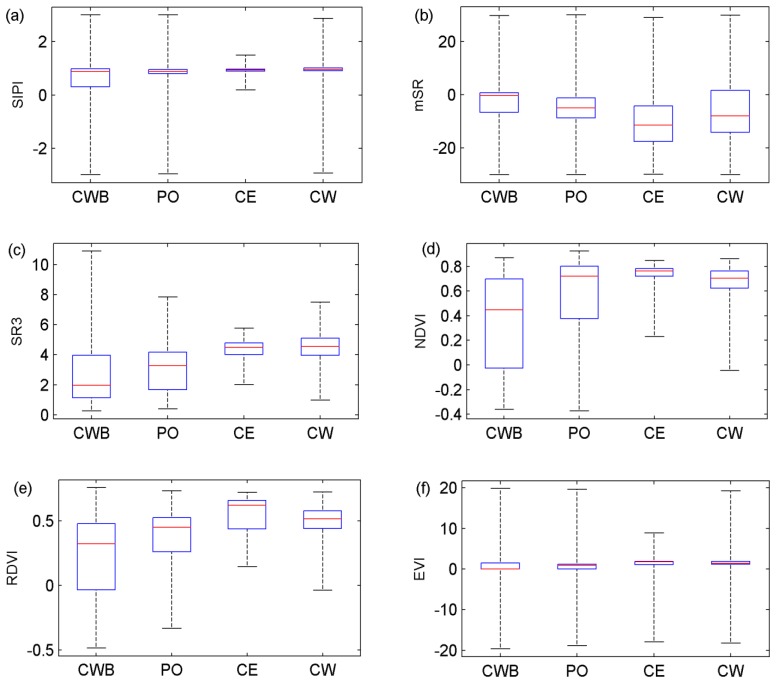
Boxplots of the VI values individually for the VI types of (a) SIPI; (b) mSR; (c) SR3; (d) NDVI; (e) RDVI and (f) EVI.

As a whole spectrum was complicated when analyzing its spatial distribution, the vegetation indices capable of featuring the typical spectral features of trees were calculated instead. The calculations were individually conducted in terms of the six vegetation indices as listed in Table 2. The statistical results are displayed in Figure 4, wherein each box aimed at a type of trees accounts for all of the trees of that type. From the comparisons of the sub-figures, it can be derived that the statistical results based on VIs, no matter leaf-level or canopy-level, present the similar trends as in Figure 3. That is, the first two tree species show larger variations in terms of all of the VIs, even though whose introduction was aimed at overcoming various known and unknown disturbing factors. In other words, accurate investigation of tree spectral reflectance still needs to locate the laser points with the corresponding VI values out of the normal range.

In fact, although VIs cannot remove all of the influences on the hyperspectral data, they still can be valid in the process of locating the points with their VI values out of the normal range in a spatial sense. Specifically, the laser points lying within the trees but corresponding to the pixels with such as NDVIs [31] less than zeros can be reckoned as the such points. The reason is that for the vegetation, the “red edge”-featured NDVI variables [31] tend to be positive and the SR3 variables [30] are generally larger than ones. In accordance to these principles, the spatial parts of the trees with vegetation indices that can manifest the real spectral reflectance characteristics of the trees can be extracted. The extracted parts as such are called the “relatively pure” (RP) parts hereafter.

### 3.3. Spatial Distributions of Vegetation Indices

**Figure 5.**
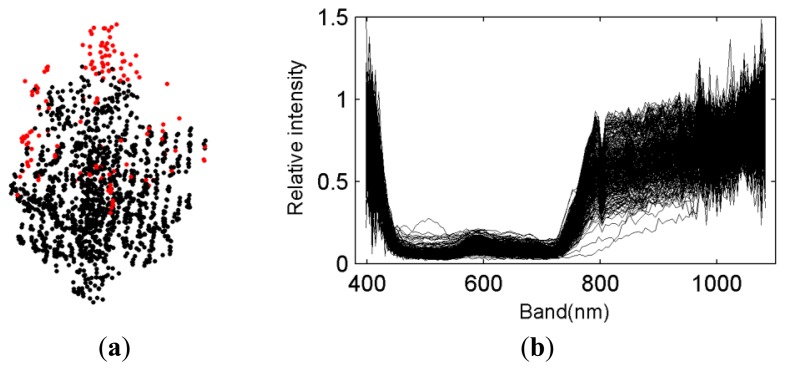
Illustrations of the spatial distributions of VIs (Red: VIs out of normal ranges) (a) and the spectra of the RP parts after processing for the same tree (b) as in Figure 3(a).

The investigation of VI spatial distributions started from the artificial specifications of VI ranges, which can show the rough distributions of various tree parts. Here, three presumptions were made, *i.e.*, NDVI being larger than zeros, SR3 being larger than ones, and the values of relative reflectance at the band of 700 nm being less than ones. The latter one was derived based on the pre-examination of the targeted trees. The resulting spatial distributions of VIs are illustrated in Figure 5a, wherein the red points mean that their related VIs are out of the prescribed normal ranges. After all of the target trees were checked, the rough inferences of the distributions of various tree parts were made. That is, the parts that are easily impacted in this study are mainly located at the top parts and the crown-edge parts of the trees.

The feasible explanations are that the top parts are influenced mainly by the direct incidence of sunlight. That is why the top parts have many echoes with relative reflectance values larger than ones. As regards the crown-edge parts, they are located at the edges of trees with low density of leaves, and thus, the collected hyperspectral pixels each comprise a large ratio of the spectra of background. This impact also exists in the top parts. With the tree parts that are easily impacted in mobile hyperspectral imaging determined, the RP parts can be found. The spectra of the RP parts are illustrated in Figure 5c. Compared to Figure 3a, the resulting spectra after the procedure of VI-based tree partition can better characterize the spectral reflectance of the trees. As the determination of RP parts is based on VIs mainly involving the visible spectral bands, the large spectral variations caused by the scattering effect at the short-wave infrared bands still exist, as shown by the variations of reflectance over 800 nm in Figure 5c.

**Figure 6.**
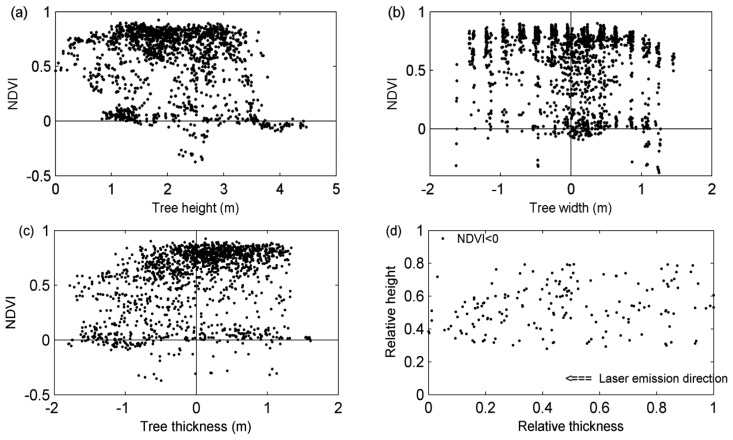
Illustration of the NDVI values along with tree height (a), tree width (b), and tree thickness (c) for a PO tree. (d) shows the relative spatial distribution of all of the points with NDVI < 0 lying in the scanned half-crowns for the 18 PO trees.

In order to better understand the characteristics of tree spectral reflectance, a rough partition is not enough and the spatial distributions of vegetation indices need to be quantitatively analyzed. With the frame of the rough tree parts as the reference, the quantification of VI spatial distributions was conducted in terms of three variables, *i.e.*, the height of the laser point, its distance to the central laser scanning profile and its distance to the central vertical plane perpendicular to the laser scanning profile. With the three parameters introduced, the locations of the points with the spectra of interest in the tree spaces can be explicitly determined, and then, the spatial variations of the VI values can be represented in a relatively continuous mode. As illustrated in Figure 6, the NDVI values along with point heights and their distances to the central laser scanning profiles can be listed for different tree species.

From Figure 6, it can be derived that with NDVI values less than zeros, the points with the heights larger than 3.5 m in Figure 6a, with the widths close to zero in Figure 6b and with the thickness across zero in Figure 6c correspond to the top parts defined beforehand. At the same time, with NDVI values less than zeros, the points with the heights less than 3.5 m in Figure 6a, with the widths larger than ones in Figure 6b and with the thickness across zero in Figure 6c relate to the crown-edge parts. In this way, the desired RP parts can be acquired in the pattern of not block-size parts but point coordinates or even space representation functions. Then, the spectral reflectance characteristics of the trees can be depicted in a quantitative way, and their mathematical forms can be induced from Figure 6. For the tree in illustration, its normal NDVI values can be characterized as a function with constant dependent variables for all of the independent variables. Specifically, the NDVI values can be reckoned to keep constant for both the height of the laser point and its distance to the central laser scanning profile. If better accuracies are demanded, the NDVI values can alternatively be fitted with a second-order function, e.g., NDVI = -0.05 *w^2^* + 0.8 in Figure 6b (*w* means the distances between the points and the central scan profile), or a Gaussian function. The fitting functions need to be chosen by users in accordance to the specific technical demands in various practical applications.

From Figure 5a, it can be found that there are still several red points lying in the central parts of the scanned half-crowns. This is also evidenced by Figure 6a–c, namely, some points with NDVI values less than zeros are out of the top and crown-edge parts. The points lying in the central parts of the scanned half-crowns were studied as follows. The points under consideration are restricted into the space with 20%–80% tree height and 20%–80% tree width, which can decrease the affects of the top and crown-edge parts. Then, the resulting points are projected onto the central scan profile plane, with tree thickness and tree height as the X and Y coordinate variables. With the laser scanning data capable of indicating their locations, such points for all of the PO trees are shown in Figure 6d. The “relative” in Figure 6d is defined by dividing the related variables against their maxima respectively. Note that four trees show no such points. From Figure 6d, it can be learnt that the points with NDVI values less than zeros are distributed almost in a random mode, no matter at the front or back half crowns. In other words, the generation of such spectral reflectance is hard to track in a stable manner, or at least based on the line spectrometer and laser scanner with relatively low resolutions used in this study.

Overall, a more comprehensive grasp of the spatial characteristics of tree spectral reflectance needs to assume mobile terrestrial line spectrometers with higher spatial resolutions, mobile laser scanners with higher sampling densities and positioning accuracies, or even hyperspectral laser scanners [36] in the next-step work.

### 3.4. Spectral Reflectance Characteristics for Different Tree Species

**Figure 7.**
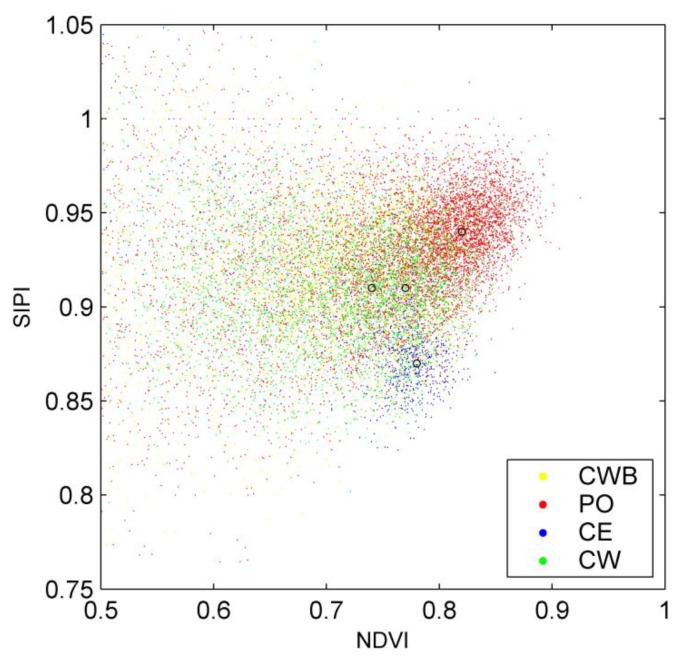
Illustration of tree spectral reflectance characteristics for different tree species based on NDVI and SIPI. The circles indicate the clustering centers respectively.

After the parts of the trees with relatively pure spectra have been located, the spectral reflectance characteristics for different tree species can be compared. Figure 7 illustrates the spectral reflectance characteristics of trees with NDVI and SIPI as the variables. Note that all of the trees in each tree type were accounted for in Figure 7. It can be learnt that the four tree species each have their own clustering centers in the NDVI-SIPI feature space. Particularly for the PO and CE tree species, they have obvious deviations. In other words, although the moderate or even low sampling density of the used mobile laser scanner cannot help solve the puzzle of spectral mixing in the pixel level (partly indicated by the large number of the feature-pair points with NDVI value less than 0.65), it is still useful for improving the investigation of tree spectral reflectance characteristics. The resulting spectra have the potential for tree species classification, which will be exploited in the next work.

## 4. Conclusion

The evaluations have validated the feasibility that mobile terrestrial hyperpsectral imaging and laser scanning can be applied for investigation of tree spectral reflectance characteristics. Although the line spectrometer and laser scanner assumed in this study present relatively low sampling resolutions, the parts of the trees with large spectral reflectance variations can be located. Based on vegetation indices, the spatial distributions of the spectra with variations at different levels can further be quantitatively depicted for the point cloud of each tree. With the crown parts that can demonstrate the real spectral reflectance characteristics of trees extracted, the properties of trees can be retrieved more accurately. The spectral reflectance characteristics for different tree species can also be shown in the VI-composed feature space. Overall, with the inferences of this study incorporated, mobile terrestrial hyperspectral imaging and laser scanning is potential for more comprehensive and accurate forestry applications.

## Figures and Tables

**Table 1.** The tree species in this study and the numbers of trees per species.

**Index**	**Common Name**	**Latin Name**	**Number**
1	Common Whitebeam (CWB)	*Sorbus aria*	8
2	Pedunculate Oak (PO)	*Quercus robur*	18
3	Camperdown Elm (CE)	*Ulmus glabra camperdownii*	5
4	Crack Willow (CW)	*Salix fragilis*, ‘*Bullata*’	8

**Table 2.** The typical VIs selected for the assessment of tree spectral reflectance.

**Scale**	**Index**	**F.**	**Reference**
Leaf	SIPI (Structure Insensitive Pigment Index)	(1)	Penuelas *et al.*, 1995 [28]
	mSR (Modified Simple Ratio)	(2)	Sims and Gamon, 2002 [29]
	SR3 (Simple Ratio 3)	(3)	Gitelson and Merzlyak, 1997 [30]

Canopy	NDVI (Normalized Difference VI)	(4)	Tucker, 1979 [31]
	RDVI (Renormalized Difference VI)	(5)	Roujean and Breon, 1995 [32]
	EVI (Enhanced VI)	(6)	Huete *et al.*, 1997 [33]
